# The Stimulatory Effects of Nanochitin Whisker on Carbon and Nitrogen Metabolism and on the Enhancement of Grain Yield and Crude Protein of Winter Wheat

**DOI:** 10.3390/molecules24091752

**Published:** 2019-05-06

**Authors:** Yingying Cheng, Yi Wang, Yanlai Han, Dongya Li, Zhongkui Zhang, Xueqiang Zhu, Jinfang Tan, Hezhong Wang

**Affiliations:** 1 College of Resources and Environmental Science, Henan Agricultural University, Zhengzhou 450002, Henan, China; yycheng93@163.com (Y.C.); wangyi19830705@163.com (Y.W.); lidongya8576@163.com (D.L.); zhangzhongkui@hotmail.com (Z.Z.); zhuxueqiang126@126.com (X.Z.); tanjf@henau.edu.cn (J.T.); 2 State Key Laboratory of Wheat and Maize Crop Science, Zhengzhou 450002, Henan, China; 3 Collaborative Innovation Center of Henan Grain Crops, Zhengzhou 450002, Henan, China; 4 College of Plant Protection, Henan Agricultural University, Zhengzhou 450002, Henan, China

**Keywords:** nanochitin whisker, dry matter, nitrogen, accumulation, translocation, metabolic enzymes, metabolism, winter wheat, yield and crude protein

## Abstract

Nanochitin whisker (NC) with a cationic nature could enhance plant photosynthesis, grain yield, and quality of wheat, but have not been systematically studied. This study was designed to investigate the stimulatory effects of NC on dry matter (DM) and nitrogen (N) accumulation and translocation, and on the metabolism of carbon (C) and N in later growth stages of winter wheat to reveal the enhancement mechanism of grain yield and crude protein concentration. Different parts of NC-treated plants from pot grown experiments were collected at the pre- and post-anthesis stages. The accumulation, translocation, and contributions of DM and N from pre-anthesis vegetation organs to grains, as well as key metabolic enzyme activities, including sucrose phosphate synthase (SPS) and phosphoenolpyruvate carboxylase (PEPC), were examined. The results showed that, at an application rate of 6 mg·kg^−1^ of NC in the soil, the accumulation of DM and N were significantly enhanced by 16.2% and 38.8% in pre-anthesis, and by 15.4% and 30.0% in post-anthesis, respectively. Translocation of N and DM in the post-anthesis periods were enhanced by 38.4% and 50.9%, respectively. NC could also stimulate enzyme activities, and increased 39.8% and 57.1% in flag leaves, and by 36.0% and 58.8% in spikes, respectively, at anthesis. SPS and PEPC increased by 28.2% and 45.1% in flag leaves, and by 42.2% and 56.5% in spikes, respectively, at 15 days after anthesis. The results indicated that the NC promoted N metabolism more than C metabolism, and resulted in the enhancement of grain yield by 27.56% and of crude protein concentration in grain by 13.26%, respectively.

## 1. Introduction

Metabolism of carbon (C) and nitrogen (N) is the most basic metabolic process in plants [1]. The accumulation and translocation of both plant dry matter and N are closely related to crop yield and important quality indicators [2,3]. Due to the low efficiency of fertilizers applied to cropping systems, N limitations may greatly inhibit future carbon fixation [4]. To improve crop and grain quality, the regulation of the accumulation and translocation of dry matter and N in crop production has attracted great attention around the world. Different approaches, such as fertilization, water regulation, cultivation and tillage, variety utilization, and application of exogenous regulators, have been implemented to enhance the accumulation and translocation of dry matter and N to boost crop production [5,6,7]. Some nanoparticles and amino acids have been used as plant growth biostimulants to enhance wheat yield and grain quality [8,9,10,11].

Plant growth and development are not only dependent on the absorption of carbon, nitrogen, and other essential elements for photosynthesis, but are also related to interdependence between C- and N-metabolism. Sucrose phosphate synthase (SPS), phosphoenolpyruvate carboxylase (PEPC), glutamine synthetase (GS), and nitrate reductase (NR) are important metabolic enzymes in N assimilation, anaplerotic C flow, and sucrose synthesis in the leaves of many higher plant species [12,13]. The coordination of C and N flow into amino acids and carbohydrates can be achieved by regulation of crucial enzyme activities through several mechanisms [14]. It has been reported that the activity of SPS, a key enzyme in the sucrose biosynthetic pathway, was rapidly and reversibly modulated by light signal and dark signal [15]. Plant SPS plays a key role in photosynthetic carbon metabolism, nevertheless, N metabolites were required for the synthesis and activation of PEPC and SPS. In other words, C metabolites are necessary for the synthesis and activation of enzymes catalyzing the assimilation of N [16].

Chitin, the second most abundant polysaccharide on earth, is composed of N-acetylglucosamine oligosaccharide monomer. Chitosan and chitooligosaccharide, derivatives of chitin, have been studied for their effects on crop growth promotion, C or N metabolism, stress resistance, and plant disease defense elicitation [17,18,19]. Application of nano-chitosan associated NPK fertilizer could significantly increase wheat yield and grain quality, stimulate orchid seed germination and protocorm development, and enlarge the chloroplast size of dendrobium leaves [20]. Moreover, it have been extensively reported in previous studies that chitin and its derivatives could regulate C and N metabolism in wheat seedlings and enhance plant resistance to biotic or abiotic stresses, including drought, plant diseases, and insect pests [19,21,22,23]. Furthermore, chitosan could induce the accumulation of indole acetic acid (IAA), salicylic acid (SA), and jasmonic acid (JA) in *Arabidopsis* root, the plant hormones regulating plant growth and development [24]. The biological activity of chitin or its derivatives is attributed to its degree of deacetylation, polymerization or molecular weight, oligomer structure, charge density, application rates, and application methods [25,26].

In our previous studies, we found that cationic nanochitin whisker when applied at an optimal rate could inhibit root rot diseases of tobacco and wheat [18,27], promote photosynthesis, and enhance grain yield of winter wheat by promoting net photosynthesis rate, stomatal conductance, intercellular CO_2_ concentrations, and transpiration rate in flag leaf at the grain-filling stage [28]. Grain protein, iron, and zinc contents in wheat were also increased dramatically when treated with nanochitin. However, the effects of nanochitin whisker on the translocation of dry matter and N, and metabolism of C and N in the later growth stages of wheat have not been reported. In this study, we explored the stimulatory effects of nanochitin whisker on the accumulation and translocation of dry matter and N, as well as on C and N metabolism during later growth stage of winter wheat, to reveal the mechanism of nanochitin whisker-enhancing grain yield and crude protein concentration in grain. This will provide a significant advancement in future sustainable agriculture, food, and natural resource systems.

## 2. Results

### 2.1. Characteristics of Nanochitin Particle

**Figure 1:**
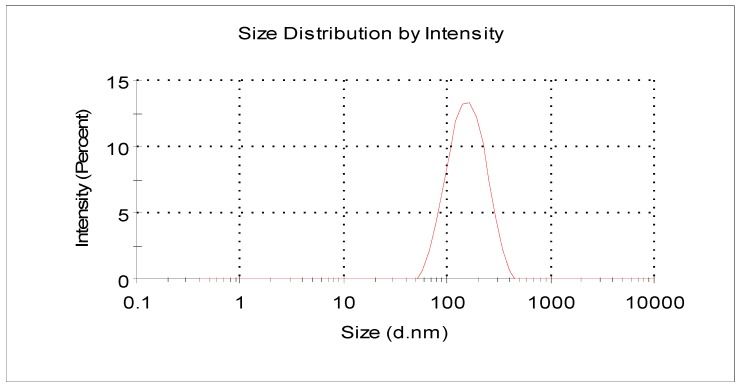
Particle size and size distribution of nanoparticles examined by DLS. A nanochitin sample, with a concentration of 0.0024% *w*/*v*, was prepared by diluting a stock of nanochitin suspension with DI water, then 1 mL of the dilution was examined by NanoSizer, ZS 90, and the pH of the dilution was not adjusted. The polydispersity index (PDI) was 0.246.

**Figure 2:**
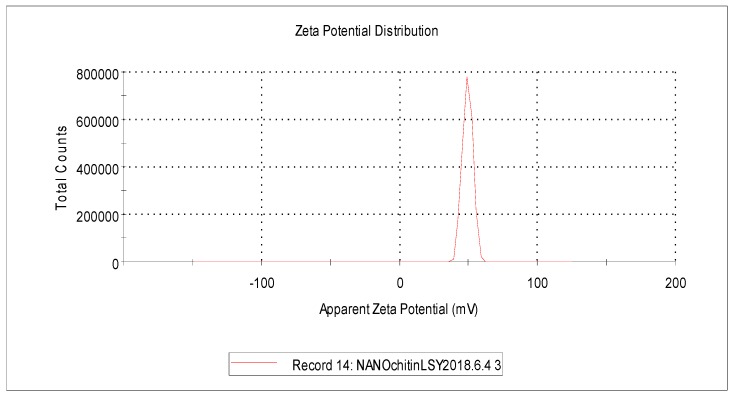
The zeta potential of nanochitin particles in DI water, which was determined by DLS. The value of the zeta potential of the nanochitin was +49.4 mV.

**Figure 3:**
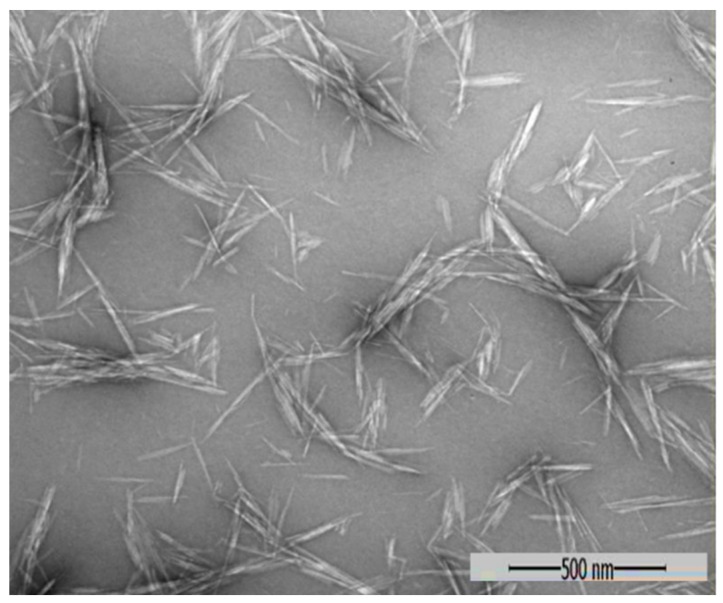
Morphological characteristics of nanochitin whisker visualized by TEM. A nanochitin sample with a concentration of 0.0024% *w*/*v* was prepared by diluting a stock of nanochitin suspension with DI water. Some of the nanoparticles were aggregated during the drying process of the sample in preparation for TEM.

The characteristics, including size and size distribution, zeta potential, and morphology of the nanochitin particles, were characterized by DLS and transmission electron microscopy (TEM,) respectively. The results showed that the final product of the nanoparticle was positively charged, with an average hydrodynamic diameter of 138.9 nm (Figure 1), and a zeta potential of +49.4 mV (Figure 2). The morphology of the particles visualized by TEM appeared as whiskers. This spindle-like structure was in the range of 15–30 nm in width and 100–250 nm in length (Figure 3). The difference in size measured by different methods was mainly based on the fundamental principles of the analytical technique and physicochemistry of the nanoparticle in solution [29]. Each technique has its own advantages and limitations. Because of its hygroscopicity in an aqueous solution and inherent hydrophilicity, the average hydrodynamic diameter of nanochitin in solution was larger than those of the particles visualized under electron microscopy. A positive value of the zeta potential reveals the nanochitin’s cationic nature in aqueous solution [30].

### 2.2. Effects of Nanochitin on Grain Yield and Crude Protein Concentration

The yield and protein concentration are the most important factors for wheat production, which are governed by the accumulation and translocation of dry matter and nitrogen. In this study, the grain yield and crude protein concentration of the winter wheat treated with nanochitin first rose and then dropped with the increase in nanochitin application rate, because the number of spikes, grains per spike, and kernel weight (1000 grain weight) were all enhanced with the same dynamic trend according to the application rate of nanochitin in soil (Table 1). Compared with the 0 mg·kg^−1^, the treatments with 2 mg·kg^−1^ and 6 mg·kg^−1^ of nanochitin in the soil significantly increased grain yield and protein concentration in wheat, in which the grain yield was increased by 19.14% and 27.56%, respectively, and the crude protein concentration was increased by 9.92% and 13.26%, respectively, which are in agreement with our previous report [28]. However, when the application rate of nanochitin was increased to 20 mg·kg^−1^, the nanochitin had less effects on the yield components of wheat, even though the kernel weight still increased slightly at this application rate. When the 20 mg·kg^−1^ application rate was compared to the 0 mg·kg^−1^ rate, there were no significant changes in the number of spikes, the number of grains per ear, yield, and the crude protein concentration in grain.

### 2.3. Enhancement of Dry Matter and N Accumulation by Nanochitin

The grain yield and quality could be affected by dry matter and nitrogen accumulation in winter wheat in pre- and post-anthesis. Post-anthesis describes the accumulation of nutrients during the period from flowering to maturity. The total dry matter accumulation at anthesis (DMA-A) and at maturity (DMA-M) in this study were enhanced first and then dropped with the increase in the nanochitin application rate, and reached a peak rate of 6 mg·kg^−1^ of nanochitin (Table 2). Compared with the control, both treatments of 2 mg·kg^−1^ and 6 mg·kg^−1^ of nanochitin significantly enhanced DMA-A by 11.23% and 16.15%, and DMA-M by 10.67% and 16.01%, respectively. However, the nanochitin rate of 20 mg·kg^−1^ had less effect on the dry matter accumulation. The dynamic feature of dry matter accumulation in the post-anthesis period (DMA-PA) was the same as those of DMA-A and DMA-M. From Table 1, we can also see that at the 2 mg·kg^−1^ and 6 mg·kg^−1^ nanochitin application rate, the DMA-PA was increased by 7.68% and 15.35%, respectively, while applying 20 mg·kg^−1^ nanochitin had less effect on DMA-PA.

The total N accumulations in winter wheat treated with nanochitin at anthesis (NA-A) and at maturity (NA-M) were increased first and then decreased with the increase in the nanochitin application rate as well. Compared with the control, the treatment rates of 2 mg·kg^−1^ and 6 mg·kg^−1^ nanochitin significantly increased NA-A by 26.21% and 38.83%, and NA-M by 25.20% and 37.40%, respectively. When the nanochitin application rate reached 20 mg·kg^−1^, N accumulation had no significant enhancement when compared to the control. The effect of the nanochitin application rate on N accumulation in the post-anthesis period (NA-PA) was almost the same as N accumulation at anthesis (NA-A) and mature (NA-M) periods. However, the NA-PA under 2 mg·kg^−1^ and 6 mg·kg^−1^ nanochitin application rates was increased by 20.00% and 30.00% respectively, but the nanochitin application rate at 20 mg·kg^−1^ only enhanced the N accumulation by 15.00%.

### 2.4. Improvement in the Retranslocation of Dry Matter and N from Pre-Anthesis Vegetation Organs to Grains

During plant growth, dry matter and N are translocated to grain from previous accumulation in the vegetative organs during anthesis to maturity stage. In this study, we found that nanochitin could also enhance dry matter accumulation in the vegetative organs at maturity (DMA-VO-M), the dry matter translocation (DMT), and percentage of translocation from pre-anthesis vegetative organs to grains (DMTP), as well as the N accumulation in vegetative organs at maturity (NA-VO-M), translocation of N (NT), and percentage of N translocated from pre-anthesis vegetative organs to grains (NTP), and reached a peak at the 6 mg·kg^−1^ of nanochitin application rate (Table 3).

Compared with the control, both treatments of 2 mg·kg^−1^ and 6 mg·kg^−1^ of nanochitin significantly enhanced DMT by 31.12% and 38.35%, respectively, and DMTP by 3.90 percentage points and 4.17 percentage points, respectively. However, the nanochitin rate of 20 mg·kg^−1^ had less effects on DMT and DMTP. A similar trend was found for NT and NTP when treated at different rates of nanochitin. As Table 2 shows, the 2 mg·kg^−1^ and 6 mg·kg^−1^ of nanochitin application rates significantly increased NT by 36.36% and 50.91%, respectively, and NTP by 4.07 percentage point and 4.17 percentage point, respectively. When the nanochitin application rate reached 20 mg·kg^−1^, the NT and the NTP were all below those of 0 mg·kg^−1^.

### 2.5. Effects of Nanochitin on Contributions of Dry Matter and N to Grain

Both the accumulations of grain dry matter and N are derived from two processes: translocation of vegetative organs in pre-anthesis and the new accumulation in the post-anthesis period. The contributions of dry matter (CR-DMT) and N translocation (CR-NT) from the pre-anthesis vegetative organs to grain, as well as the accumulation of dry matter (CR-DMA) and N (CR-NA) in the post-anthesis period to grain, reflect the relative importance of the sources of dry matter or N in grain, respectively. However, with nanochitin application rate increase, the dynamic trends of the CR-DMA and CR-NA response to nanochitin rate were opposite to that of CR-DMT and CR-NT.

Compared with the control, the CR-DMA under the 2 mg·kg^−1^ and 6 mg·kg^−1^ of nanochitin application rates significantly decreased by 4.85 percentage points and 4.49 percentage points, respectively, and the CR-NA decreased by 2.16 percentage points and 2.72 percentage points, respectively (Table 4). When the nanochitin application rate reached 20 mg·kg^−1^, neither CR-DMA nor CR-NA had a significant difference between the treatment and control. 

### 2.6. Effects of Nanochitin on the Ratio of N to Dry Matter Accumulation and Translocation

**Figure 4:**
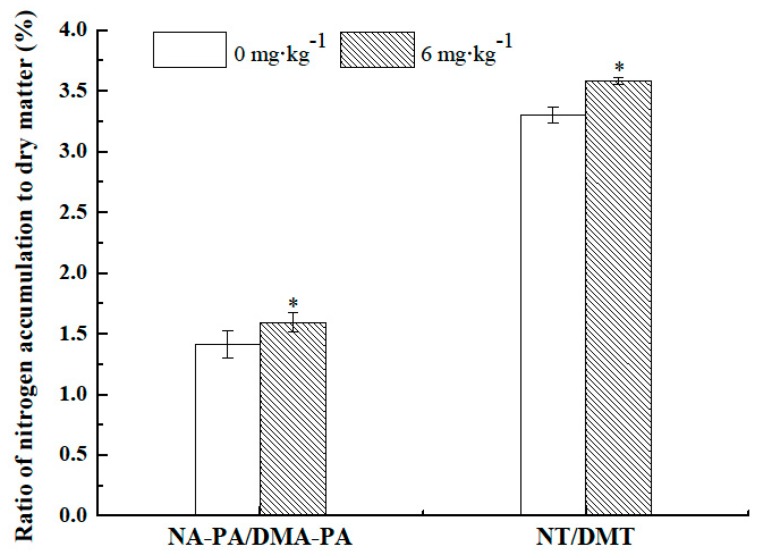
Effects of nanochitin on the ratio of N accumulation to dry matter in post-anthesis and transported from pre-anthesis vegetative organs. NA-PA: the N accumulation in post-anthesis period, DMA-PA: the dry matter accumulation in post-anthesis period, NT: the N translocation, and DMT: the dry matter translocation. Error bar represents significant differences at *p ≤* 0.05.

N concentration in dry matter at different growing stages was important for crops to get high yield and protein content in grain. The ratio of N to dry matter accumulation in the post-anthesis period (NA-PA/DMA-PA) and the ratio of N to dry matter translocation from pre-anthesis vegetative organs (NT/DMT) in wheat both increased when treated with nanochitin. Figure 4 shows that, when compared with 0 mg·kg^−1^, nanochitin at an optimal application rate of 6 mg·kg^−1^ significantly enhanced the ratios of NA-PA/DMA-PA and NT/DMT by 12.77% and 8.48%, respectively. The results of higher enhancement of the ratio of NA-PA to DMA-PA than that of NT to DMT indicated that the appropriate rate of nanochitin not only promoted the accumulation of nitrogen and dry matter in both the pre- and post-anthesis growth stages and increased the translocation of dry matter and nitrogen, but also the accumulation and translocation of N was predominately promoted more than that of dry matter.

### 2.7. Regulation of Carbon and Nitrogen Metabolism

To understand the mechanism of the enhancement of yield and quality, carbon and N metabolism were key factors to explore. In this study, we investigated two important metabolic enzymes, SPS and PEPC, and how their activities were stimulated by nanochitin. From Table 5, we can see that the treatment rate of 6 mg·kg^−1^ of nanochitin significantly enhanced the activities of SPS and PEPC at the anthesis, which increased SPS and PEPC in the flag leaves by 39.75% and 57.09%, and 35.97% and 58.79% in spikes, respectively. At 15 days after the anthesis of the wheat, nanochitin applied at a rate of 6 mg·kg^−1^ increased the SPS and PEPC in the flag leaves by 28.20% and 45.08%, respectively, and 42.23% and 56.52% in spikes, respectively. The results from enzymatic studies showed that nanochitin could simultaneously improve C and N metabolism in flag leaves and spikes during the anthesis and grain filling stages of wheat. 

The ratio of SPS/PEPC indicated the degree of enhancement in C metabolism when N was strengthened. Compared with the control, applying 6 mg·kg^−1^ nanochitin decreased the ratio of SPS to PEPC in flag leaves and spikes at the anthesis and the 15-days after anthesis stages of winter wheat. Among them, the decline in flag leaves and spikes were 11.11% and 14.58% at anthesis, and 11.69% and 8.45% at 15 days after anthesis, respectively. The results showed that nanochitin promoted nitrogen metabolism more than carbon metabolism.

## 3. Discussion

C allocation might be an important prerequisite for plant growth in an N-rich environment [31]. Deficiency of N may greatly inhibit future carbon fixation [32]. C and N metabolism is accomplished based on the increases in accumulation and translocation of both dry matter and N in wheat for grain filling and crude protein synthesis. It has been reported that chitin had a low C/N ratio and was beneficial to the proliferation of soil microorganisms when applied in soil [33]. Microbial degradation of chitin in soil contributes substantially to carbon cycling and release in terrestrial ecosystems, and the latter could easily establish a symbiotic relationship with plant mycorrhizal fungi or rhizobium and promote nutrient absorption, especially for N fixation [34,35]. In a previous study, we found that cationic nanochitin whisker could boost photosynthesis and grain yield for both large spike and multi-spike varieties of winter wheat [28]. This project was a continuation of previous studies and mainly focused on the effects of nanochitin whisker on the absorption process and translocation of dry matter and N, as well as C and N metabolism at pre- and post-anthesis stages of wheat.

The results showed that applying nanochitin whisker at an appropriate dosage in the soil could promote the accumulation of dry matter and N in wheat, both at pre- and post-anthesis stages (Table 2), which was in agreement with the previous study [28]. In this study, we found that nanochitin whisker promoted wheat N uptake at dosages lower than 6 mg·kg^−1^ in soil. This phenomenon was attributed to nanochitin whisker’s unique properties, such as its spindle-like structure, nano-scale size, cationic property, and high surface area [20,28]. Chitin or chitosan have biological activity only when they are produced in low molecular weight or less than 6–8 mer [19,36]. Nano chitosan, on the other hand, is commonly produced by cross-linking with sodium tripolyphosphate to form spherical or irregular particles [37], which is larger in diameter than nanochitin crystallite whisker synthesized by acidic hydrolysis. The size of nanochitin used in this study was in the range of 15–30 nm in width and 100–250 nm in length (Figure 1 and Figure 3), which is more advanced to penetrate the cell membrane when compared with bulk material of chitin [18,27,28]. As a novel bionanomaterial and low-dose effect, we noticed that, when the application rate of nanochitin was at 20 mg·kg^−1^, nanochitin whisker had less of an effect on the dry matter and/or N accumulation in pre- and post-anthesis, and the yield components of wheat [28,38].

We speculated that the effects of nanochitin on the accumulation and translocation of dry matter and N might be partially dose dependent. Its cationic property, with a zeta potential of +49.4 mv (Figure 3), and special whisker morphology (Figure 4) allow the nanoparticle to enter plant cells easily. Our results showed that the effect of the nanochitin application rate on dry matter and N accumulation differed between the pre- and post-anthesis periods. In comparison to 0 mg·kg^−1^, 2 mg·kg^−1^ and 6 mg·kg^−1^ of nanochitin whisker in the soil increased the dry matter accumulation at anthesis by 11.2% and 16.12%, and the N accumulation by 26.2% and 38.8%, respectively (Table 2). However, increases in dry matter accumulation were only by 7.7% and 15.4%, and the N accumulation by 20.0% and 30.0%, respectively, in the post-anthesis period. The effects of nanochitin on dry matter and N accumulation before anthesis were significantly higher than those after anthesis. The reasons might be as follows: (1) when nanochitin whisker was applied to the soil, it might have degraded into monomers by the chitinase secreted by microorganisms in soil over time [33] and, therefore, its effects were reduced after anthesis; (2) along with the development of seedling growth, the senescence of the root might have reduced its ability to absorb nanochitin whisker or its degraded monomers [39]; and (3) the physiological activity of nanochitin whisker absorbed at early stages might also be reduced due to biodegradation in plants [35,40].

The substances stored in vegetative organs are one of the important sources for grain yield and nitrogen accumulation. In this study, the appropriate dosage for nanochitin whisker application simultaneously increased the quantities of nutrients translocated from pre-anthesis vegetative organs to grains (Table 3). It was generally considered that the retranslocation of nutrients in pre-anthesis vegetative organs was affected by many factors, such as grain demand for nutrients, especially N, source-sink relationships, nutrient supply levels after anthesis, and plant senescence process [41,42]. Among these, the degradation of macromolecules, such as proteins and phloem transport, are the key regulatory points, related to sophisticated regulatory networks [43]. Therefore, further exploration of the mechanism of nanochitin regulating the transport of stored substances in pre-anthesis vegetative organs of crops is necessary and in progress.

Accumulation of dry matter and N in grain is derived from two processes: translocation from pre-anthesis vegetative organs and from new accumulation after the post-anthesis period to grains. Enhancing translocation of dry matter and N from pre-anthesis vegetative organs would provide a material basis and would guarantee the coordinated improvement of grain yield and protein yield [44,45]. In nanochitin-treated plants, the contributions of dry matter and N to translocation from pre-anthesis vegetative organs to grains, rose first and then dropped, while the percentage of dry matter and N contributed from the accumulation in the post-anthesis period to grains showed the opposite trend. The optimal application rate of nanochitin promoted the retranslocation, more than the accumulation, of dry matter and N in the pre-anthesis vegetative organs (Table 4). Therefore, nanochitin could be functionalized as a plant growth promotor to stimulate plant growth, as an alternative for crop management in the pre-anthesis stage to enlarge plant vegetative organs and increase grain yield and protein content.

The improvements in grain yield and N concentration are significant for crops to achieve high yield and good quality. Generally, yield and protein concentration for gramineous crops, such as wheat, often show a negative correlation [46]. But our results showed that the grain yield and protein concentration increased simultaneously under an optimal application rate of nanochitin whisker in soil (Table 1). This might be attributed to the synergistic promotion of accumulation and translocation of dry matter and N in crops, on the one hand, and might also be related to increases in the ratio of accumulation of N to dry matter in post-anthesis and the translocation of N to dry matter from pre-anthesis vegetative organs (Figure 4). Most importantly, nanochitin could predominately promote the accumulation and translocation of N rather than for dry matter.

Previous studies showed that coordinating the metabolic balance between C and N is the key process to improving both the grain yield and N concentration [47,48], because C and N metabolism compete for the C skeleton and reduce energy generated by photosynthesis. Both SPS and PEPC played important roles in both C and N metabolism and regulating the distribution ratio of the C skeleton between C and N metabolism, which controlled the direction of plant metabolism [12]. SPS could promote crop dry matter accumulation by facilitating greater C skeleton transformation to carbohydrates, while PEPC could affect protein synthesis by enhancing more C skeleton distribution to amino acid biosynthesis. Yu et al. [49] reported that the relative activities of PEPC and SPS decided the carbohydrate transformation direction between decomposition and synthesis, and that higher SPS/PEPC could relatively enhance the ability of carbon metabolism when nitrogen metabolism was strengthened. Chitin oligosaccharides affected the expression of genes related to C and N metabolism in wheat seedlings [19,36]. We found that nanochitin could both improve the activities of SPS and PEPC, but reduced the ratios of SPS to PEPC in flag leaves and spikes during the flowering and grain-filling stages (Table 5), which suggested that nanochitin promoted nitrogen metabolism more than carbon metabolism in wheat. Nanochitin whisker might play an important role in improving both plant C and N metabolism by stimulating metabolic enzymes expression and activities. The stimulating characteristics of nanochitin on SPS and PEPC activities and the ratio of SPS to PEPC may better explain the reason why an optimal application rate of nanochitin can coordinately enhance wheat yield and grain crude protein concentration.

## 4. Materials and Methods

### 4.1. Reagents

Chitin (from shrimp shell, practical grade, coarse flakes) was purchased from Sigma Aldrich (Sigma-Aldrich, St. Louis, MO, USA). Hydrochloric acid (HCl) (36–38%), sulfuric acid (H_2_SO_4_, 98.08%), and hydrogen peroxide (H_2_O_2_, 30%) were purchased from Zhengzhou Shenghe Instruments Co., Ltd. (Zhengzhou, Henan, China). The water used in the experiment was deionized (DI) water from the Millipore Direct-Q 5 ultrapure water system. All other chemicals used in the nanochitin preparation were analytical grade.

### 4.2. Preparation and Characterization of Nanochitin

A nanochitin suspension was obtained by acidic hydrolysis following the method described by Xue et al. [28]. Briefly, after hydrolysis, the product was dialyzed against DI water in a regenerated cellulose dialysis tube to remove hydrochloric acid residue until the pH remained consistent (about 6.2) in the dialysis system. The final nanochitin suspension was sonicated for 15 min in an ice bath at an output of 35% with a 500 W ultrasonic processor (model VC-505, Sonics & Materials, Inc., Newtown, CT, USA), followed by a serious filtration using PVDF syringe filters of 1.0, 0.45, and 0.22 µm, subsequently. The Z-average hydrodynamic diameter (cumulant mean) and zeta potential of the nanochitin particles were determined using dynamic light scattering (DLS). Measurements were performed in triplicate at 25 °C using a Malvern Zetasizer Nano ZS90 (Malvern Panalytical Ltd., Malvern, UK) with 90° scattering optics. The sample (0.0024%, *w*/*v*) was diluted with DI water from stock solution of the nanochitin suspension without adjustment of the pH or ionic strength. For morphology of the particles, a drop of the nanochitin dilution was stained with a 2% uranyl acetate negative stain on a carbon-coated copper grid (200 meshes). The sample was visualized by a JEOL transmission electron microscope (JEM 1400) (JEOL USA, Inc., Peabody, MA, USA) with an accelerating voltage of 80 kV.

### 4.3. Plant Materials and Treatments

The pot experiment was conducted in the winter wheat growing season at the experimental station at Henan Agricultural University, Zhengzhou, China. A multi-spike variety of winter wheat of Zhou Mai 32 was used in this study. The soil was fluvo-aquic soil with medium texture, collected from the top layer of the planting area at the North China Scientific Observation and Experimental Station for Nutrition and Fertilization of Wheat/Maize Rotation, Ministry of Agriculture, Xuchang County, Henan province, China. The basic physical and chemical properties of the soil included a concentration of 13.07 g·kg^−1^ of organic matter in soil, 0.98 g·kg^−1^ of total N, 19.34 mg·kg^−1^ of available P, and 108.52 mg·kg^−1^ of available K. The pH of the soil was 7.32.

Plastic pots, 30 cm × 30 cm in size, were employed and accommodated 10 kg of air dried soil for the test. During the test period, the pots were placed outdoors and arranged randomly with frequent position changes. Based on a previous study for dose response [28], four rates of 0, 2, 6, and 20 g·kg^−1^ of nanochitin in the soil were set in this experiment as a comparison. Each pot received the same amount of fertilizer with pure 0.15 g·kg^−1^ N, 0.2 g·kg^−1^ P_2_O_5,_ and 0.1 g·kg^−1^ K_2_O. All P_2_O_5_ and K_2_O were applied as a starter. N was applied as starter by 2/3 and as a topdressing by 1/3 at the jointing stage. Twelve wheat seeds were planted in each pot at approximately a 2 cm sowing depth. After seedling setting, 2 L of nanochitin suspension, based on the designed application rate, was applied to each corresponding pot. DI water served as a control. During the growth period, general management for pest control and irrigation were conducted as necessary. All experiments were carried out in triplicate for each sampling.

### 4.4. Fresh Sample Treatments and Assay of Enzymes

At anthesis and maturity, the above ground parts of the plants were sampled and divided into different parts by organs. The fresh samples were dried at 105 °C for 30 min, then continuously dried at 65 °C and weighed at 2h intervals until the weight remained unchanged in a period of 24 h. After crushing and sieving through a 60 mesh, the samples were digested by H_2_SO_4_-H_2_O_2_, and the nitrogen content was determined using a Type AA3 continuous flow analyzer.

The metabolic key enzymes, including sucrose phosphate synthase (SPS) and phosphoenolpyruvate carboxylase (PEPC), were extracted and measured for flag leaves and spikes at the flowering stage and 15 days after grain filling. The flag leaves and ears of wheat at flowering and filling stage were selected randomly. The activities of SPS and PEPC were determined following the method described by Wang and Huang [50]. In detail, for SPS measurement, about 1 g of wheat sample was mixed with 3 mL of the extraction medium, containing 100 mmol·L^−1^ Tris-HCl buffer (pH 7.2), 10 mmol·L^−1^ MgCl_2_, 1 mmol·L^−1^ EDTA, 10 mmol·L^−1^ β-Mercaptoethanol, 2% ethylene glycol, and 1% pyrrolidone in a pre-cooled mortar and rapidly ground. The homogenate was centrifuged at 15,000× *g*, at 4 °C for 10 min and then the supernatant was collected for the measurement. About 0.4 mL of reaction buffer, containing 100 mmol·L^−1^ Tris-HCl (pH 7.2), 10 mmol·L^−1^ MgCl_2_, 5 mmol·L^−1^ UDPG, and 5 mmol·L^−1^ fru-6-P, was mixed with the supernatant and incubated first at 30 °C for 30 min, and then in boiling water for 5 min. After adding 0.1 mL of 2 mmol·L^−1^ NaOH, the reaction liquid was further boiled for 10 min. About 1 mL of 0.1% resorcinol and 3.5 mL of 30% HCl were then added into the reaction and incubated at 80 °C for 10 min. When the reaction finished, the mixture was cooled down and absorbance was measured using a spectrophotometer (UV-1600, Shimadzu, Tokyo, Japan) at 480 nm wavelength.

For PEPC measurement, about 0.5 g of wheat sample was mixed with 3 mL of pre-cooled 100 mmol·L^−1^ Tris-HCl buffer, containing 5% glycerol, 1% PVP, 1 mmol·L^−1^ EDTA, and 7 mmol·L^−1^ mercaptoethanol, at pH 8.2 in a pre-cooled mortar. After rapid grinding, the homogenate was centrifuged at 15,000× *g*, at 4 °C for 20 min and the supernatant was collected for measurement. About 1 mL of reaction buffer, containing 50 mmol·L^−1^ Tris-HCl (pH 7.8), 10 mmol·L^−1^ MgCl_2_, 0.25 mmol·L^−1^ EDTA, 5.0 mmol·L^−1^ NaHCO_3_, 2.0 mmol·L^−1^ DTT, 4 U MDH, 0.1 mmol·L^−1^ NADH, and 2.0 mmol·L^−1^ PEP, was added and incubated at 28 °C for 10 min. Then, about 200 μL of PEPC extraction solution was added into the reaction. The absorbance at 340 nm wavelength was measured spectrophotometrically.

### 4.5. Calculations of Relevant Indicators

The following formulas were adopted from Chen et al. [51] for the calculation of the relevant indicators:

Grain crude protein concentration (g·kg^−1^) = grain N concentration (g·kg^−1^) × 5.83 and (1)

DMA-PA (or NA-PA) = DMA-M (or NA-M) - DMA-A (or NA-A),(2)

where, DMA-PA: dry matter accumulation in the post-anthesis period, DMA-M: total dry matter accumulation at maturity, DMA-A: total dry matter accumulation at anthesis, NA-PA: N accumulation in the post-anthesis period, NA-M: total N accumulation at maturity, and NA-A: total N accumulation at anthesis (unit: g·pot^−1^).

DMT (or NAT) = DMA-A (or NA-A) - DMA-VO-M (or NA-VO-M),(3)

where, DMT: dry matter translocation to grains from pre-anthesis vegetative organs, DMA-A: total dry matter accumulation at anthesis, DMA-VO-M: dry matter accumulation in vegetative organs at maturity, NT: N translocation to grains from pre-anthesis vegetative organs, NA-A: total N accumulation at anthesis, and NA-VO-M: N accumulation in vegetative organs at maturity (unit: g·pot^−1^).

DMTP (or NTP) = DMT (or NT) × 100/DMA-A (or NA-A),(4)

where, DMTP: the percentage of dry matter translocation from pre-anthesis vegetative organs to grains, DMT: dry matter translocation to grains from the pre-anthesis vegetative organs, DMA-A: total dry matter accumulation at anthesis, NTP: the percentage of N translocation from the pre-anthesis vegetative organs to grains, NT: N translocation to grains from the pre-anthesis vegetative organs, and NA-A: total N accumulation at anthesis (unit: g·pot^−1^).

CR-DMA-PA (or CR-NA-PA) = DMA-PA (or NA-PA) ×100/DMA-G-M (or NA-G-M),(5)

where, CR-DMA-PA: the contribution of dry matter accumulation in post-anthesis period to grains, DMA-PA: dry matter accumulation in the post-anthesis period, DMA-G-M: dry matter accumulation in grains at maturity, CR-NA-PA: the contribution of N accumulation in the post-anthesis period to grains, NA-PA: N accumulation in the post-anthesis period, and NA-G-M: N accumulation in grains at maturity (unit: g·pot^−1^).

CR-DMT (CR-NT) = DMT (NT) ×100/DMA-G-M (NA-G-M),(6)

where, CR-DMT: the contribution of dry matter translocation from the pre-anthesis vegetative organs to grains, DMT: dry matter translocation to grains from the pre-anthesis vegetative organs, DMA-G-M: dry matter accumulation in grains at maturity, CR-NT: the contribution of N translocation from the pre-anthesis vegetative organs to grains, NT: N translocation to grains from the pre-anthesis vegetative organs, and NA-G-M: N accumulation in grains at maturity (unit: g·pot^−1^).

### 4.6. Data Analysis

Variance analysis and multiple comparison for the data were conducted using SPSS 22.0 software (IBM, New York, NY, USA). Differences at *p* < 0.05 were considered to be statistically significant.

## 5. Conclusions

Nanochitin whisker at an application rate of 6 mg·kg^−1^ in soil could significantly promote dry matter and N accumulation, and translocation from vegetative organs to grains in post-anthesis, which could result in the considerable enhancement of grain yield and crude protein concentration in grain. This is also attributed to the stimulation of key metabolic enzymes, SPS and PEPC activities, in flag leaves and spikes. As a green nanobiomaterial, there is a great potential for the enrichment of crop production and the reduction of chemical fertilizer use in future agriculture sustainability.

## Figures and Tables

**Table 1:** Effects of nanochitin on grain yield and crude protein concentration of winter wheat.

C_Nanochitin_* (mg·kg^−1^)	Number of Spike	Grains/Spike	Kernel Weight	Yield	Crude Protein Content (g·kg^−1^)
	(number·pot^−1^)	(number·ear^−1^)	(g·1000 grain^−1^)	(g·pot^−1^)	
0	31 ± 1.53b^**^	30 ± 0.81b	34.35 ± 0.22c	27.43 ± 0.57c	142.18 ± 6.44b
2	34 ± 0.58a	31 ± 0.31a	36.29 ± 0.59b	32.68 ± 0.83b	156.28 ± 4.44a
6	35 ± 1.00a	32 ± 0.72a	37.32 ± 0.15a	34.99 ± 0.08a	161.03 ± 3.15a
20	30 ± 1.00b	29 ± 0.67b	34.88 ± 0.51c	26.26 ± 0.68d	138.46 ± 7.75b

* C_Nanochitin_, Nanochitin application rate (mg·kg^−1^). ** Different letters of a, b, c, and d after the same column of data show significant differences among different treatments (*p* ≤ 0.05).

**Table 2:** Enhancements in dry matter and N accumulation in pre- and post-anthesis of wheat.

C_Nanochitin_* (mg·kg^−1^)	DMA-A (g·pot^−1^)	DMA-M (g·pot^−1^)	DMA-PA (g·pot^−1^)	NA-A (g·pot^−1^)	NA-M (g·pot^−1^)	NA-PA (g·pot^−1^)
0	77.05 ± 2.30c^**^	91.00 ± 2.58c	13.94 ± 0.47c	1.03 ± 0.07c	1.23 ± 0.09c	0.20 ± 0.02b
2	85.70 ± 2.04b	100.71 ± 1.97b	15.01 ± 0.44b	1.30 ± 0.06b	1.54 ± 0.07b	0.24 ± 0.02a
6	89.49 ± 2.57a	105.57 ± 2.45a	16.08 ± 0.23a	1.43 ± 0.06a	1.69 ± 0.07a	0.26 ± 0.01a
20	72.47 ± 1.96c	85.18 ± 1.89d	12.71 ± 0.29d	0.92 ± 0.05c	1.09 ± 0.07c	0.17 ± 0.02c

* C_Nanochitin_: nanochitin application rate (mg·kg^−1^), DMA-A: the total dry matter accumulation at anthesis, DMA-M: the total dry matter accumulation at maturity, DMA-PA: the dry matter accumulation in post-anthesis period, NA-A: the N accumulation at anthesis, NA-M: the N accumulation at maturity, and NA-PA: the N accumulation in post-anthesis period. ** Different letters of a, b, c, and d after the same column of data show significant differences among different treatments (*p* ≤ 0.05).

**Table 3:** Effects of nanochitin on dry matter and N retranslocation from pre-anthesis vegetation organs to grains.

C_Nanochitin_* (mg·kg^−1^)	DMA-VO-M (g·pot^−1^)	DMT (g·pot^−1^)	DMTP (%)	NA-VO-M (g·pot^−1^)	NT (g·pot^−1^)	NTP (%)
0	60.31 ± 0.95c**	16.74 ± 1.37c	21.70 ± 1.15b	0.48 ± 0.01c	0.55 ± 0.05b	53.70 ± 1.82b
2	63.75 ± 1.02b	21.95 ± 1.21b	25.60 ± 0.86a	0.55 ± 0.02b	0.75 ± 0.04a	57.77 ± 0.68a
6	66.33 ± 1.50a	23.16 ± 1.08a	25.87 ± 0.47a	0.60 ± 0.02a	0.83 ± 0.04a	57.87 ± 0.37a
20	58.26 ± 1.12c	14.21 ± 0.93c	19.60 ± 0.81c	0.45 ± 0.01c	0.47 ± 0.03c	50.58 ± 1.18c

* C_Nanochitin_: nanochitin application rate (mg·kg^−1^), DMA-VO-M: the dry matter accumulation in vegetative organs at maturity, DMT: the dry matter translocation, DMTP: the percentage of dry matter translocation from pre-anthesis vegetative organs to grains, NA-VO-M: the N accumulation in the vegetative organs at maturity, NT: the N translocation, and NTP: the percentage of N translocated from pre-anthesis vegetative organs to grains. ** Different letters of a, b, c, and d after the same column of data show significant differences among different treatments (*p* ≤ 0.05).

**Table 4:** Effects of nanochitin on the contribution rates of dry matter and N in wheat grain.

C_Nanochitin_* (mg·kg^−1^)	CR-DMT	CR-DMA	CR-NT	CR-NA
	(%)	(%)	(%)	(%)
0	54.51 ± 1.88b**	45.49 ± 1.88a	73.73 ± 1.20b	26.27 ± 1.20a
2	59.36 ± 1.82a	40.64 ± 1.82b	75.89 ± 1.00a	24.11 ± 1.00b
6	59.00 ± 1.37a	41.00 ± 1.37b	76.45 ± 0.72a	23.55 ± 0.72b
20	52.76 ± 2.06b	47.24 ± 2.06a	72.84 ± 0.84b	27.16 ± 0.84a

* C_Nanochitin:_ application rate of nanochitin (mg·kg^−1^), CR-DMT: the contribution of dry matter translocated from pre-anthesis vegetative organs to grains, CR-DMA: the contribution of dry matter accumulated in the post-anthesis period to grains, CR-NT: the contribution of N translocated from the pre-anthesis vegetative organs to grains, and CR-NA: the contribution of N accumulated in the post-anthesis period to grains. ** Different letters of a, b, c, and d after the same column of data show significant differences among different treatments (*p* ≤ 0.05).

**Table 5:** Effects of nanochitin on enzymes activities for C and N metabolism at the anthesis and the 15 days after anthesis stages of winter wheat.

Organ	C_Nanochitin_* (mg·kg^−1^)	Anthesis Stage			15-Days After Anthesis		
		SPS	PEPC	SPS/PEPC	SPS	PEPC	SPS/PEPC
		(mg/g·h)	(nmol/g·min)		(mg/g·h)	(nmol/g·min)	
Flag Leaf	0	7.27 ± 0.58b^**^	10.16 ± 1.09b	0.72 ± 0.04a	4.61 ± 0.92b	5.99 ± 0.83b	0.77 ± 0.05a
	6	10.16 ± 0.89a	15.96 ± 0.74a	0.64 ± 0.03b	5.91 ± 0.43a	8.69 ± 1.12a	0.68 ± 0.05b
Spike	0	5.81 ± 1.05b	12.23 ± 1.29b	0.48 ± 0.02a	2.96 ± 0.19b	4.14 ± 0.81b	0.71 ± 0.04a
	6	7.90 ± 1.09a	19.42 ± 1.93a	0.41 ± 0.01b	4.21 ± 0.35a	6.48 ± 0.61a	0.65 ± 0.06b

* C_Nanochitin_, Nanochitin application rate (mg·kg^−1^), SPS: Sucrose phosphate synthase, and PEPC: phosphoenolpyruvate carboxylase (PEPC), SPS/PEPC: the ratio of SPS to PEPC. **Different letters of a, b, c, and d in the same column of data show significant differences among different treatments (*p* ≤ 0.05).
